# Supplementary material for: Heightened efficacy of anidulafungin when used in combination with manogepix or 5-flucytosine against Candida auris in vitro
Source: Antimicrob Agents Chemother. Author manuscript; Available in PMC 2023 Jun 26. (PMC10269148; doi:10.1128/aac.01645-22)
Supplement: Supplementary Material [file EMS176399-supplement-Supplementary_Material.pdf]

Table S1. MIC values of all tested antifungals for individual isolates.

Isolate	MIC (mg/L)					
	AMB	FLC	VRC	AFG	5FC	MGX
B12406	1	4	0.06	0.25	0.25	0.004
B15223	2	>128	16	8	0.125	0.004
**B19460**	**2**	**32**	**0.25**	**0.125**	**0.125**	**0.008**
B19547	2	32	0.25	0.125	0.125	0.004
B19617	2	32	0.125	>8	0.125	0.004
B19837	2	32	0.125	>8	0.125	0.004
**B19618**	**2**	**32**	**0.25**	**>8**	**0.125**	**0.004**
**B17040**	**2**	**>128**	**2**	**0.06**	**0.25**	**0.016**
**B17041**	**2**	**>128**	**2**	**0.125**	**0.25**	**0.03**
B17073	2	>128	2	>8	0.25	0.03
B17201	2	>128	2	>8	0.25	0.03
**B18560**	**2**	**64**	**0.25**	**0.06**	**0.125**	**0.004**
B18845	2	64	0.25	0.25	0.125	0.004
B18841	2	64	0.25	2	0.125	0.008
**B18843**	**2**	**64**	**0.5**	**0.5**	**0.125**	**0.004**
B12692	2	>128	2	0.5	0.25	0.03
**B12694**	**2**	**>128**	**2**	**0.25**	**0.125**	**0.03**
**B12663**	**2**	**>128**	**2**	**8**	**0.25**	**0.03**
**B12664**	**2**	**>128**	**2**	**0.125**	**0.25**	**0.03**
B12688	2	>128	2	>8	0.25	0.03
**B20931**	**2**	**128**	**1**	**0.06**	**0.25**	**0.008**
**B21040**	**2**	**64**	**1**	**0.03**	**0.25**	**0.004**
B21041	2	128	1	0.03	0.25	0.004
B21042	2	128	1	0.125	0.25	0.008
B21043	2	128	1	0.03	0.25	0.008

Isolates in bold were used in checkerboard assays. Resistance (red) and susceptibility (green) according to tentative CDC *C. auris* breakpoints. Grey background for inconclusive AMB MIC_{90}. 5FC, 5-flucytosine; AFG, anidulafungin; AMB, amphotericin B; FLC, fluconazole; MGX, manogepix; VRC, voriconazole

Table S2. MIC and FICI values for the combination with 5-flucytosine.

**Anidulafungin + 5-flucytosine**												
	**1st repeat**				**2nd repeat**				**3rd repeat**			
	**MIC[a] [mg/L]**				**MIC[a] [mg/L]**				**MIC[a] [mg/L]**			
**Strain**	**AFG**	**5FC**	**AFG/5FC**	**FICI[b]**	**AFG**	**5FC**	**AFG/5FC**	**FICI[b]**	**AFG**	**5FC**	**AFG/5FC**	**FICI[b]**
B19460	0.25	0.125	0.06/0.03	**0.48/SYN**	0.5	0.125	0.125/0.03	**0.49/SYN**	>0.5[c]	0.25	0.25/0.06	**0.49/SYN**
B19618	>32[c]	0.125	16/0.03	**0.49/SYN**	>32[c]	0.25	4/0.125	0.56/IND	8	0.125	0.06/0.125	1.01/IND
B17040	0.5	0.25	0.25/0.016	0.56/IND	0.125	0.25	0.008/0.0125	0.56/IND	2	0.5	0.06/0.06	**0.15/SYN**
B17041	0.25	0.25	0.06/0.125	0.69/IND	0.25	0.25	0.06/0.125	0.74/IND	0.5	0.125	0.25/0.06	0.98/IND
B18560	0.5	0.125	0.06/0.06	0.60/IND	>0.5[c]	0.125	0.06/0.03	**0.30/SYN**	>0.5[c]	0.125	0.125/0.06	0.61/IND
B18843	4	0.125	1/0.03	**0.49/SYN**	4	0.125	2/0.06	0.98/IND	4	0.125	2/0.06	0.98/IND
B12694	0.5	0.25	0.06/0.06	**0.36/SYN**	0.25	0.25	0.06/0.03	**0.36/SYN**	0.5	0.25	0.06/0.03	**0.24/SYN**
B12663	4	0.25	2/0.125	1.00/IND	32	0.25	8/0.03	**0.37/SYN**	8	0.25	4/0.06	0.74/IND
B12664	0.5	0.125	0.5/0.06	0.98/IND	0.5	0.125	0.125/0.06	0.73/IND	0.5	0.25	0.06/0.125	0.62/IND
B20931	0.5	0.25	0.06/0.016	**0.18/SYN**	0.06	0.25	0.002/0.125	0.53/IND	0.125	0.25	0.06/0.03	0.60/IND
B21040	0.06	0.25	0.001/0.25	1.02/IND	0.016	0.25	0.016/0.008	1.03/IND	0.06	0.25	0.03/0.016	0.56/IND
**Amphotericin B + 5-flucytosine**												
	**1st repeat**				**2nd repeat**				**3rd repeat**			
	**MIC[a] [mg/L]**				**MIC[a] [mg/L]**				**MIC[a] [mg/L]**			
**Strain**	**AMB**	**5FC**	**AMB/5FC**	**FICI[b]**	**AMB**	**5FC**	**AMB/5FC**	**FICI[b]**	**AMB**	**5FC**	**AMB/5FC**	**FICI[b]**
B19460	2	0.25	1/0.125	1.00/IND	2	0.5	0.016/0.5	1.01/IND	2	0.5	0.016/0.5	1.01/IND
B19618	2	0.5	1/0.008	0.52/IND	2	0.5	1/0.25	1.00/IND	2	0.5	0.03/0.5	1.02/IND
B17040	2	0.5	1/0.25	1.00/IND	2	1	0.016/1	1.01/IND	4	1	0.016/1	1.00/IND
B17041	2	0.5	1/0.25	1.00/IND	2	0.5	1/0.125	0.75/IND	2	1	1/0.5	1.00/IND
B18560	2	0.25	0.0016/0.25	1.01/IND	2	0.25	0.016/0.25	1.01/IND	2	0.5	0.016/0.5	1.01/IND
B18843	2	0.25	1/0.03	0.62/IND	2	0.5	0.016/0.5	1.01/IND	2	0.5	1/0.25	1.00/IND
B12694	2	>0.5[c]	0.125/0.5	0.56/IND	2	>0.5[c]	0.25/0.5	0.63/IND	4	>0.5[c]	2/0.5	1.00/IND
B12663	2	1	0.016/0.5	0.51/IND	2	1	0.016/1	1.01/IND	4	1	2/0.25	0.75/IND
B12664	2	0.5	1/0.125	0.75/IND	2	0.5	1/0.125	0.75/IND	4	0.5	2/0.06	0.62/IND
B20931	0.06	0.5	0.5/0.25	1.00/IND	1	0.5	0.5/0.25	1.00/IND	2	0.5	1/0.016	0.53/IND
B21040	1	0.5	0.5/0.06	0.62/IND	1	0.5	0.5/0.125	0.75/IND	1	0.5	0.5/0.25	1.00/IND
**Voriconazole + 5-flucytosine**												
	**1st repeat**				**2nd repeat**				**3rd repeat**			
	**MIC[a] [mg/L]**				**MIC[a] [mg/L]**				**MIC[a] [mg/L]**			
**Strain**	**VRC**	**5FC**	**VRC/5FC**	**FICI[b]**	**VRC**	**5FC**	**VRC/5FC**	**FICI[b]**	**VRC**	**5FC**	**VRC/5FC**	**FICI[b]**
B19460	0.5	0.125	0.004/0.125	1.01/IND	0.25	0.125	0.008/0.125	1.03/IND	0.25	0.125	2/0.125	**8.00/ANT**
B19618	0.5	0.125	0.008/0.125	1.02/IND	0.25	0.25	1/0.125	**4.50/ANT**	0.25	0.125	1/0.125	**5.00/ANT**
B17040	2	0.25	0.016/0.25	1.01/IND	2	0.25	0.016/0.25	1.01/IND	4	0.5	0.016/0.5	1.00/IND
B17041	2	0.125	0.03/0.125	1.02/IND	2	0.25	1/0.125	1.00/IND	4	0.25	2/0.016	0.56/IND
B18560	0.5	0.125	0.002/0.125	1.00/IND	0.25	0.125	1/0.06	**4.48/ANT**	0.5	0.25	0.002/0.25	1.00/IND
B18843	0.5	0.125	0.004/0.125	1.01/IND	0.5	0.25	2/0.125	**4.50/ANT**	0.5	0.25	2/0.125	**4.50/ANT**
B12694	2	0.25	0.016/0.25	1.01/IND	2	0.25	0.016/0.25	1.01/IND	2	0.25	0.016/0.25	1.01/IND
B12663	2	0.25	0.008/0.25	1.00/IND	1	0.25	0.008/0.25	1.01/IND	2	0.25	0.008/0.25	1.00/IND
B12664	2	0.125	1/0.016	0.63/IND	2	0.25	0.016/0.25	1.01/IND	2	0.25	0.016/0.25	1.01/IND
B20931	2	0.125	1/0.016	0.63/IND	1	0.125	0.008/0.125	1.01/IND	1	0.25	0.008/0.25	1.01/IND
B21040	1	0.125	0.008/0.125	1.01/IND	1	0.25	0.008/0.25	1.01/IND	1	0.25	0.008/0.25	1.01/IND

[a]90% growth inhibition endpoint for combinations with AMB. For all other combinations 50% growth inhibition endpoint was used.

[b]Minimum FICI unless antagonism was observed.

[c]MIC of single drug was rounded to the next highest two-fold dilution if 50% (90% for amphotericin B) growth inhibition was not reached at the highest concentration.

Table S3. MIC and FICI values for the combination with manogepix.

Anidulafungin + manogepix												
	1st repeat				2nd repeat				3rd repeat			
	MIC[a] [mg/L]				MIC[a] [mg/L]				MIC[a] [mg/L]			
**Strain**	**AFG**	**MGX**	**AFG/MGX**	**FICI[b]**	**AFG**	**MGX**	**AFG/MGX**	**FICI[b]**	**AFG**	**MGX**	**AFG/MGX**	**FICI[b]**
B19460	>0.5[c]	0.008	0.016/0.004	1.02/IND	0.5	0.008	0.125/0.002	**0.50/SYN**	0.5	0.008	0.125/0.002	**0.50/SYN**
B19618	8	0.004	0.125/0.004	0.52/IND	>32[c]	0.004	0.06/0.004	1.00/IND	8	0.008	0.125/0.004	0.52/IND
B17040	1	0.06	0.25/0.016	0.52/IND	0.5	0.03	0.03/0.008	**0.33/SYN**	0.5	0.03	0.008/0.016	0.55/IND
B17041	0.5	0.03	0.125/0.016	0.78/IND	0.25	0.03	0.03/0.016	0.65/IND	0.25	0.06	0.06/0.008	**0.37/SYN**
B18560	>0.5[c]	0.008	0.03/0.002	**0.28/SYN**	>0.5[c]	0.008	0.5/0.002	0.75/IND	>0.5[c]	0.008	0.125/0.0005	**0.19/SYN**
B18843	4	0.004	2/0.0005	0.63/IND	4	0.008	1/0.004	0.75/IND	4	0.008	0.125/0.004	0.53/IND
B12694	0.5	0.03	0.03/0.004	**0.19/SYN**	0.25	0.016	0.03/0.008	0.62/IND	0.5	0.03	0.125/0.008	0.52/IND
B12663	>32[c]	0.03	4/0.008	**0.33/SYN**	>32[c]	0.03	4/0.008	**0.33/SYN**	8	0.03	0.06/0.03	1.01/IND
B12664	>0.5[c]	0.06	0.03/0.016	**0.30/SYN**	0.25	0.06	0.002/0.03	0.51/IND	0.5	0.06	0.06/0.016	**0.39/SYN**
B20931	0.125	0.008	0.03/0.002	**0.49/SYN**	0.125	0.016	0.03/0.001	**0.30/SNY**	0.016	0.008	0.008/0.0005	0.56/IND
B21040	0.016	0.008	0.008/0.002	0.75/IND	0.016	0.008	0.008/0.004	1.00/IND	0.016	0.008	0.008/0.0005	0.56/IND

Manogepix + 5-flucytosine												
	1st repeat				2nd repeat				3rd repeat			
	MIC[a] [mg/L]				MIC[a] [mg/L]				MIC[a] [mg/L]			
**Strain**	**5FC**	**MGX**	**5FC/MGX**	**FICI[b]**	**5FC**	**MGX**	**5FC/MGX**	**FICI[b]**	**5FC**	**MGX**	**5FC/MGX**	**FICI[b]**
B19460	0.25	0.008	0.001/0.008	1.00/IND	0.125	0.004	0.001/0.004	1.01/IND	0.125	0.008	0.001/0.008	1.01/IND
B19618	0.25	0.004	0.125/0.002	1.00/IND	0.25	0.004	0.001/0.004	1.00/IND	0.125	0.004	0.002/0.004	1.02/IND
B17040	0.125	0.06	0.03/0.03	0.74/IND	0.25	0.03	0.002/0.03	1.01/IND	0.25	0.03	0.004/0.03	1.02/IND
B17041	0.25	0.06	0.125/0.008	0.63/IND	0.25	0.03	0.002/0.03	1.01/IND	0.25	0.06	0.125/0.03	1.00/IND
B18560	0.25	0.008	0.016/0.004	0.56/IND	0.25	0.008	0.001/0.008	1.00/IND	0.125	0.008	0.004/0.004	0.53/IND
B18843	0.25	0.004	0.125/0.016	**4.50/ANT**	0.25	0.008	0.001/0.008	1.00/IND	0.25	0.008	0.125/0.001	0.63/IND
B12694	0.25	0.03	0.001/0.03	1.00/IND	0.25	0.03	0.001/0.016	0.54/IND	0.25	0.06	0.001/0.03	**0.50/SYN**
B12663	0.25	0.03	0.002/0.03	1.01/IND	0.25	0.03	0.002/0.03	1.01/IND	0.25	0.06	0.002/0.03	0.51/SYN
B12664	0.25	0.06	0.016/0.03	0.56/IND	0.25	0.03	0.008/0.016	0.57/IND	0.125	0.06	0.06/0.016	1.01/IND
B20931	0.25	0.008	0.002/0.008	1.01/IND	0.25	0.016	0.016/0.008	0.56/IND	0.25	0.016	0.002/0.016	1.01/IND
B21040	0.25	0.004	0.001/0.004	1.00/IND	0.25	0.008	0.008/0.004	0.53/IND	0.25	0.016	0.001/0.016	1.00/IND

[a]90% growth inhibition endpoint for combinations with AMB. For all other combinations 50% growth inhibition endpoint was used.

[b]Minimum FICI unless antagonism was observed.

[c]MIC of single drug was rounded to the next highest two-fold dilution if 50% (90% for amphotericin B) growth inhibition was not reached at the highest concentration.

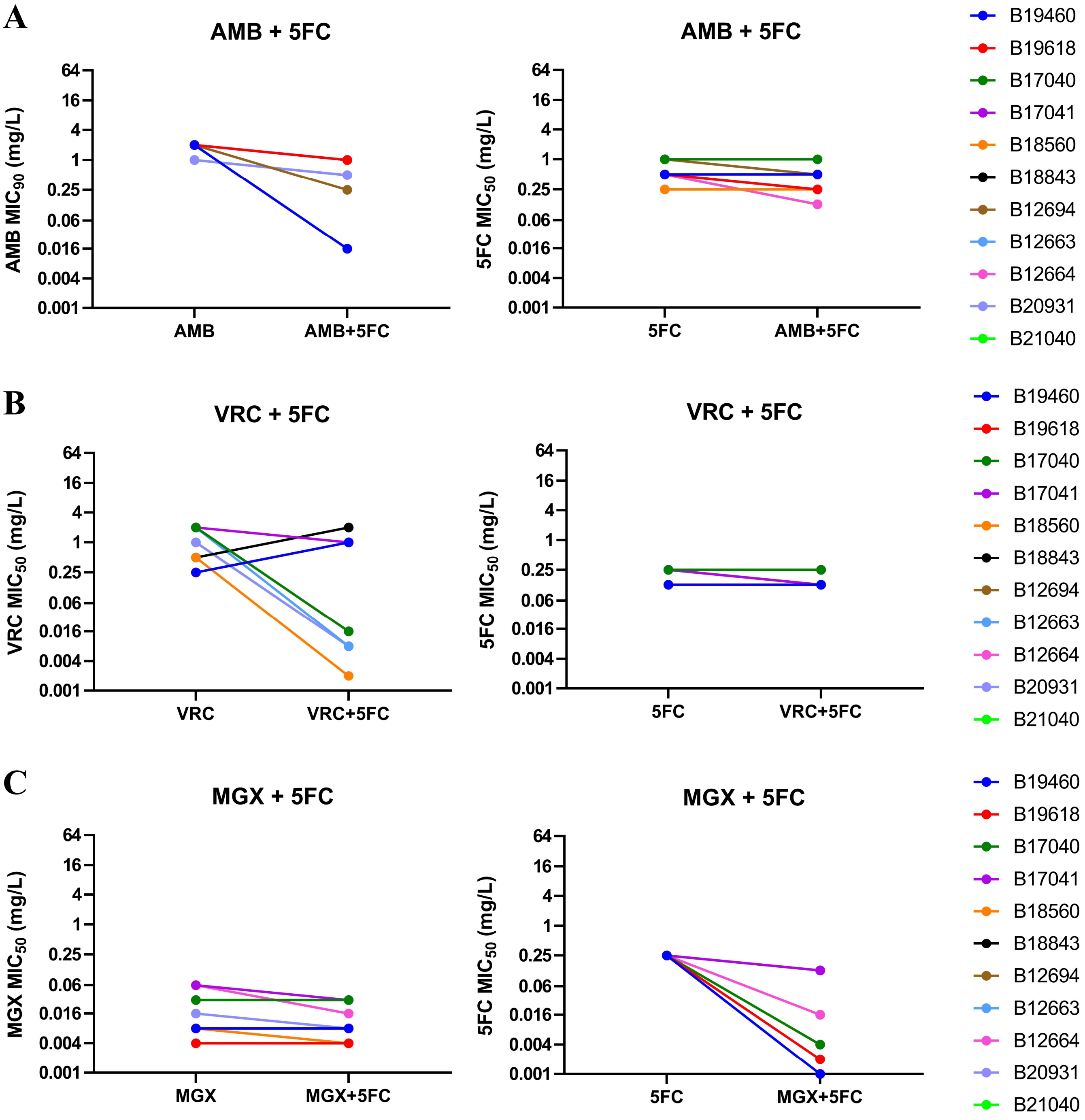

Figure S1. Changes in MIC values due to antifungal combinations for 11 *C. auris* isolates.

MIC values for 11 *C. auris* isolates in combinations of amphotericin B (A), voriconazole (B) and manogepix (C) with 5-flucytosine compared to the antifungals in monotherapy as determined by checkerboard assays. Symbols represent median values of three independent experiments. 5FC, 5-flucytosine; AMB, amphotericin B; MGX, manogepix; VRC, voriconazole.

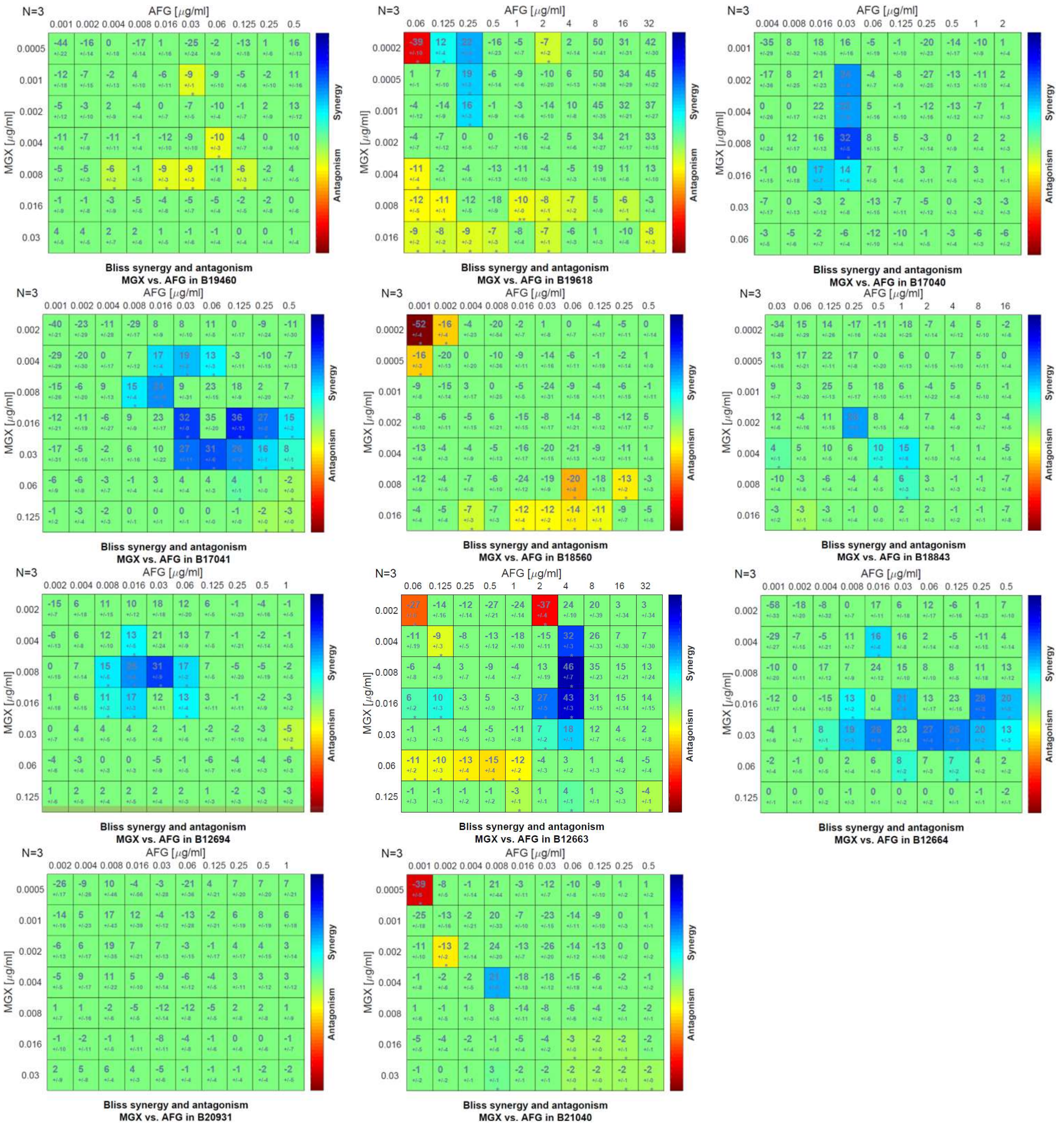

Figure S2. Matrix synergy plots for anidulafungin combined with manogepix.

The interactions of anidulafungin with manogepix were measured using checkerboard assays and analysed with Combenefit (n=3). The plots show the interaction score of each combination, the standard deviation (smaller font size) and their statistical significance (*P≤0.05; **P ≤ 0.001; ***P<0.0001. AFG, anidulafungin; MGX, manogepix.

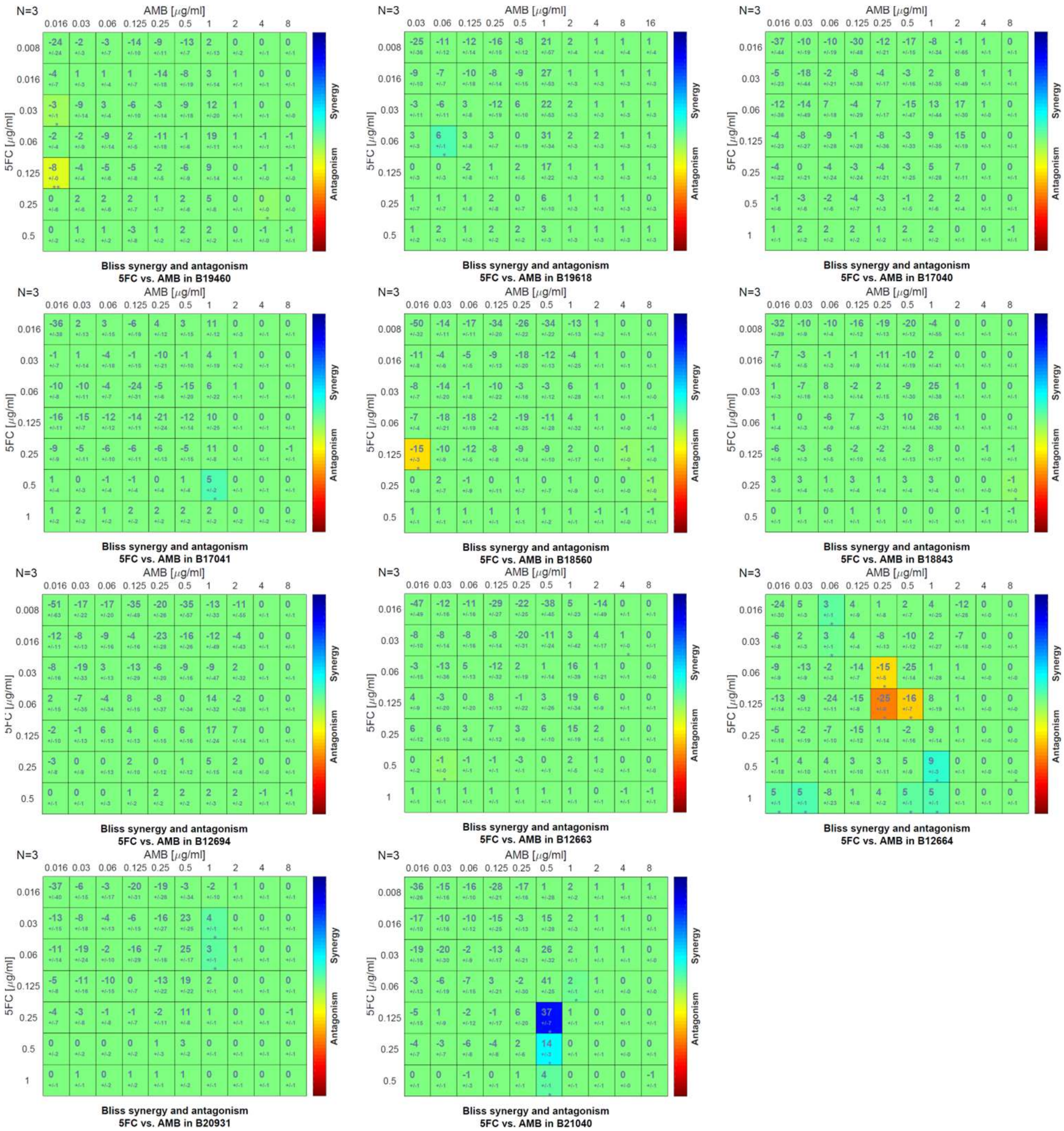

Figure S3. Matrix synergy plots for amphotericin B combined 5-flucytosine.

The interactions of amphotericin B with 5-flucytosine were measured using checkerboard assays and analysed with Combenefit (n=3). The plots show the interaction score of each combination, the standard deviation (smaller font size) and their statistical significance (*P≤0.05; **P ≤ 0.001; ***P<0.0001. 5FC, 5-flucytosine; AMB, amphotericin B.

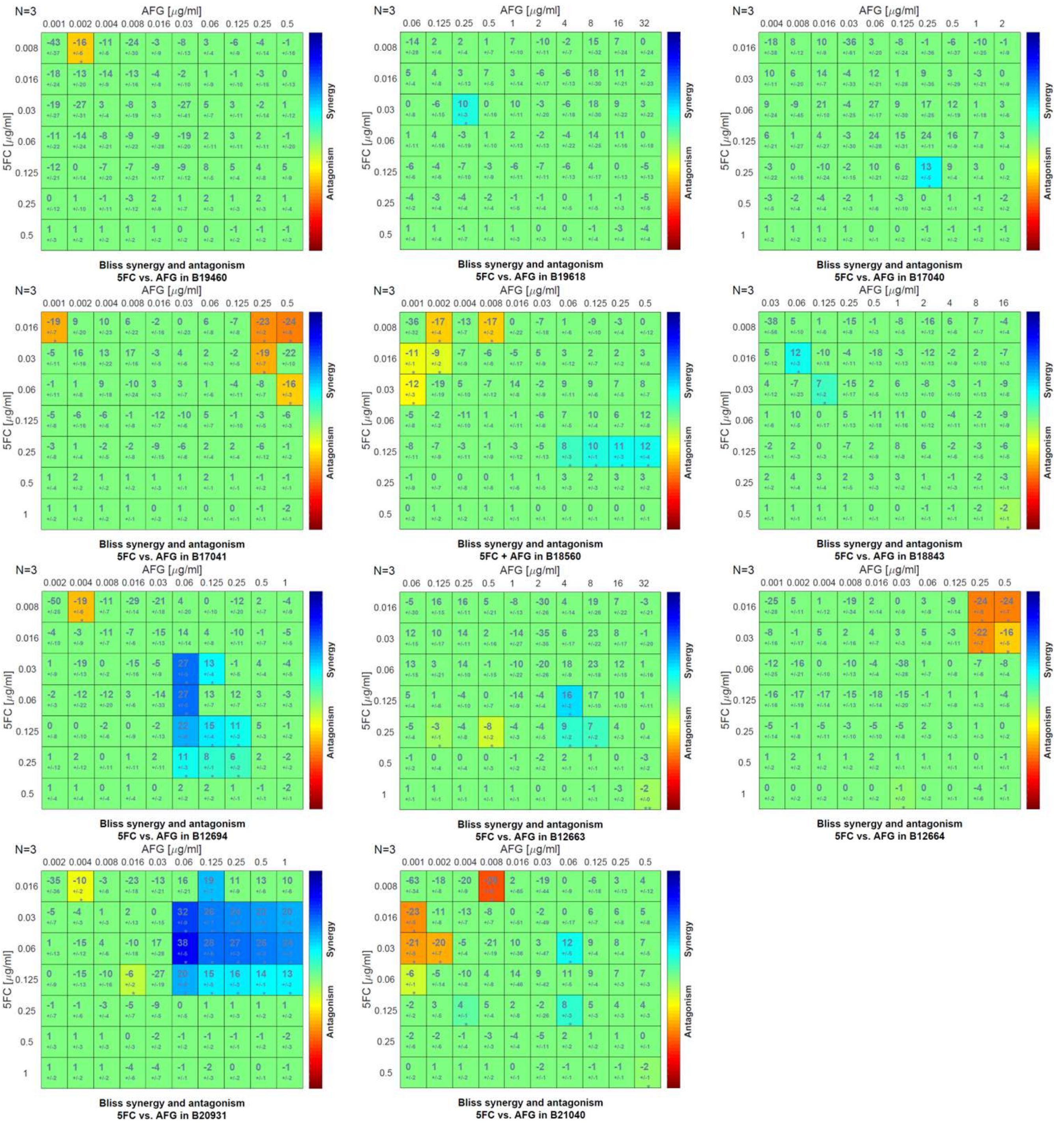

Figure S4. Matrix synergy plots for anidulafungin combined 5-flucytosine.

The interactions of anidulafungin with 5-flucytosine were measured using checkerboard assays and analysed with Combenefit (n=3). The plots show the interaction score of each combination, the standard deviation (smaller font size) and their statistical significance (*P≤0.05; **P ≤ 0.001; ***P<0.0001. 5FC, 5-flucytosine; AFG, anidulafungin.

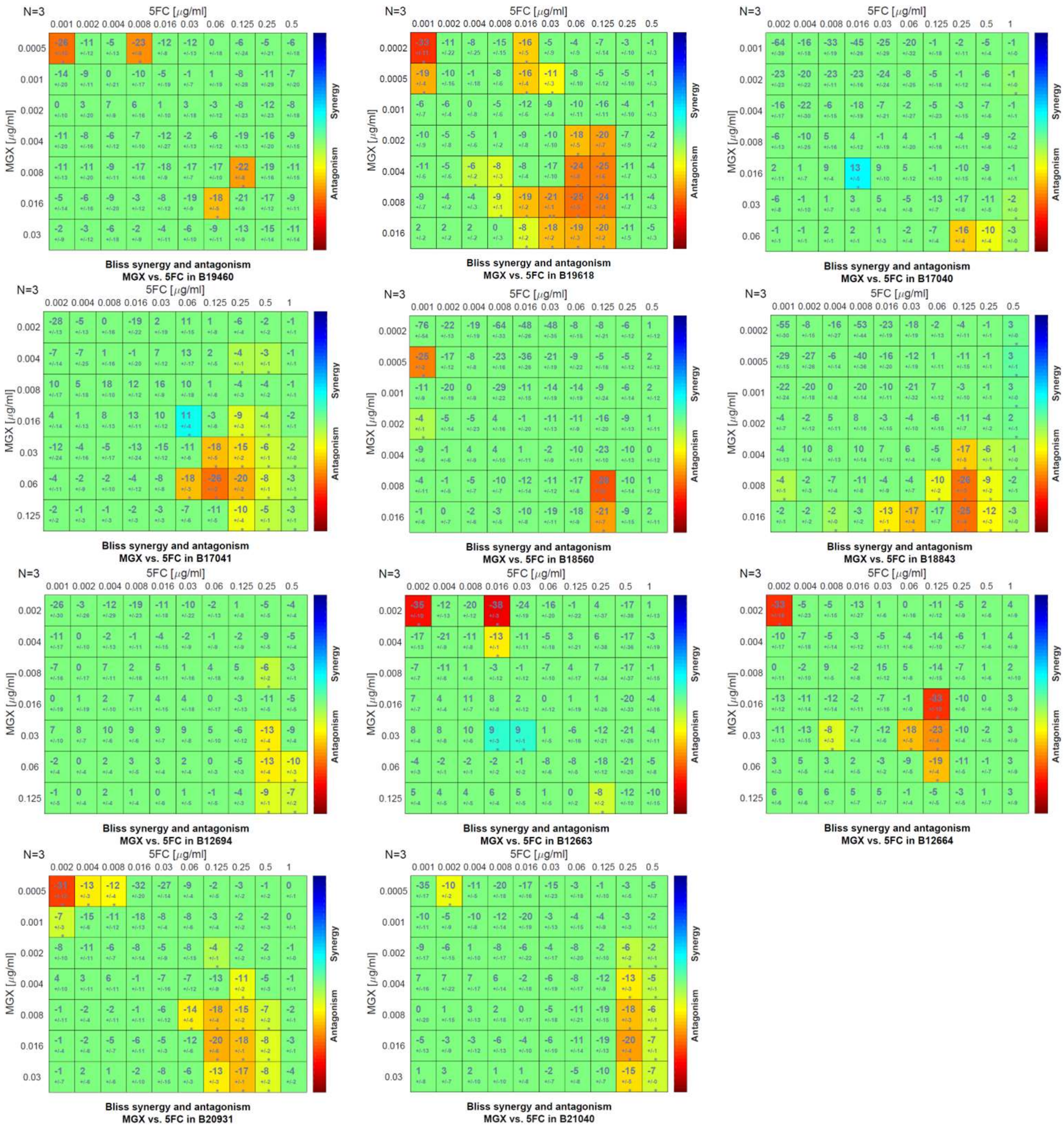

Figure S5. Matrix synergy plots for manogepix combined with 5-flucytosine.

The interactions of manogepix with 5-flucytosine were measured using checkerboard assays and analysed with Combenefit (n=3). The plots show the interaction score of each combination, the standard deviation (smaller font size) and their statistical significance (*P≤0.05; **P ≤ 0.001; ***P<0.0001. 5FC, 5-flucytosine; MGX, manogepix.

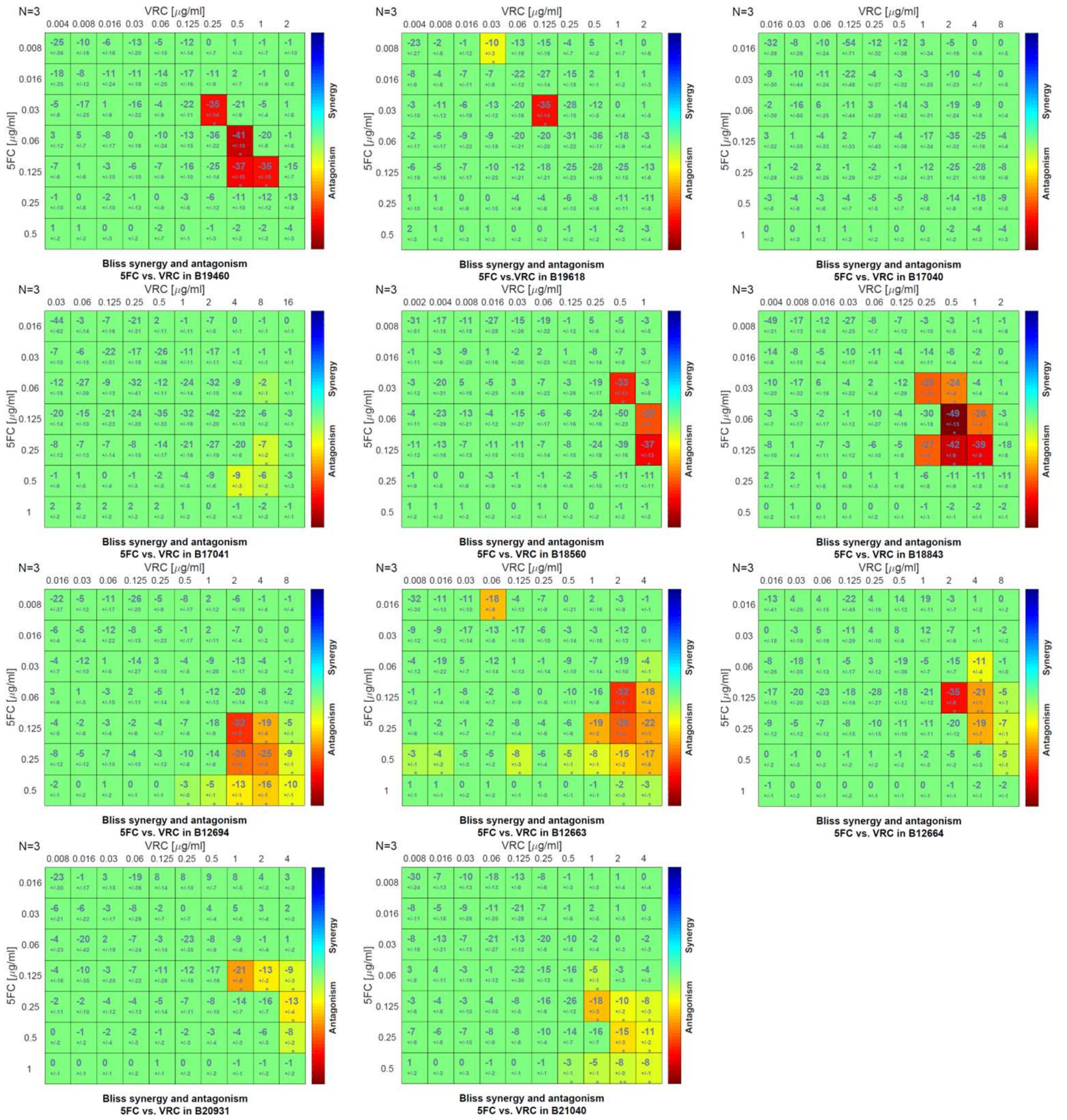

Figure S6. Matrix synergy plots for voriconazole combined with 5-flucytosine.

The interactions of voriconazole with 5-flucytosine were measured using checkerboard assays and analysed with Combenefit (n=3). The plots show the interaction score of each combination, the standard deviation (smaller font size) and their statistical significance (*P≤0.05; **P ≤ 0.001; ***P<0.0001. 5FC, 5-flucytosine; VRC, voriconazole.

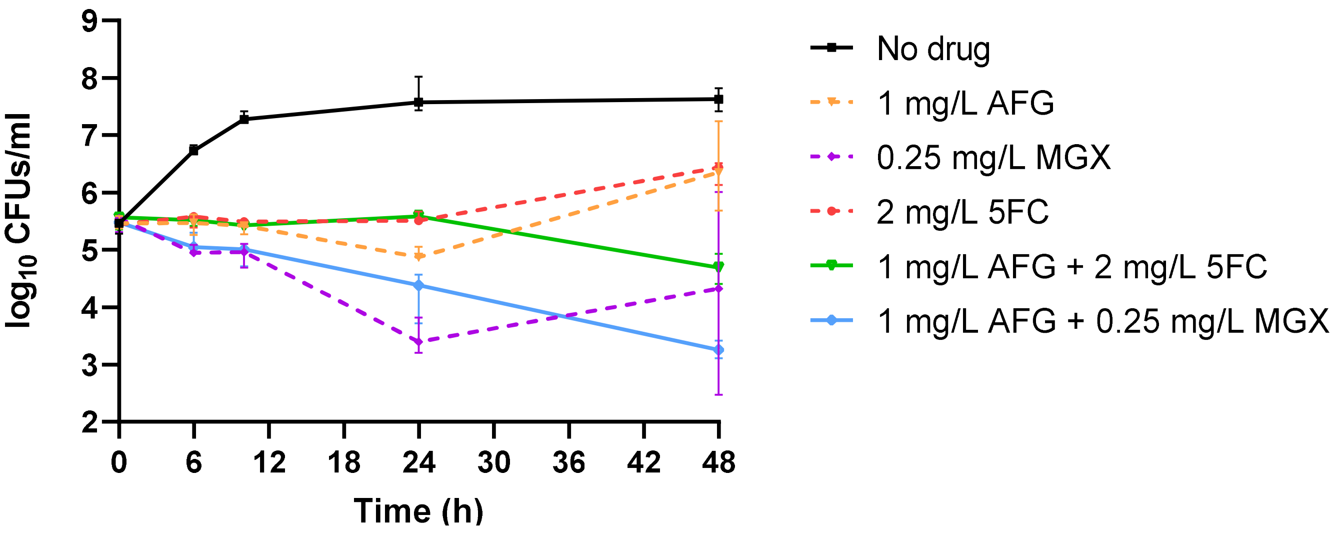

Figure S7. Time-kill curves for anidulafungin in combination with manogepix or 5-flucytosine against *C. auris*.

C. auris isolate B17041 was grown in RPMI 2%G-MOPS cultures with and without anidulafungin, manogepix or 5-flucytosine at 8x MIC_{50} in monotherapy and in combination for 48 h at 37 °C. At each timepoint, aliquots were diluted, plated onto agar plates and incubated for up to 48 h at 37 °C to determine CFUs/ml. Datapoints represent median ± range from three independent experiments. Anidulafungin, 5-flucytosine and manogepix in monotherapy showed regrowth at 48h after an initial fungistatic effect in the first 24 h. Both anidulafungin combinations achieved fungistatic activity over the complete 48 h with a reduction in CFUs/ml of 2.2 $\log_{10}$-fold and 0.8 $\log_{10}$-fold compared to the starting inoculum for combinations with manogepix and 5-flucytosine, respectively. The decline in CFUs/ml for the combination treatments is unlikely to be caused by the formation of cell aggregates as aggregation was also observed for anidulafungin and manogepix in monotherapy which did not result in a reduction in CFUs/ml. No antifungal treatment resulted in fungicidal activity, defined as ≥3 $\log_{10}$ reduction in CFU/ml compared to the starting inoculum. 5FC, 5-flucytosine; AFG, anidulafungin; MGX, manogepix.

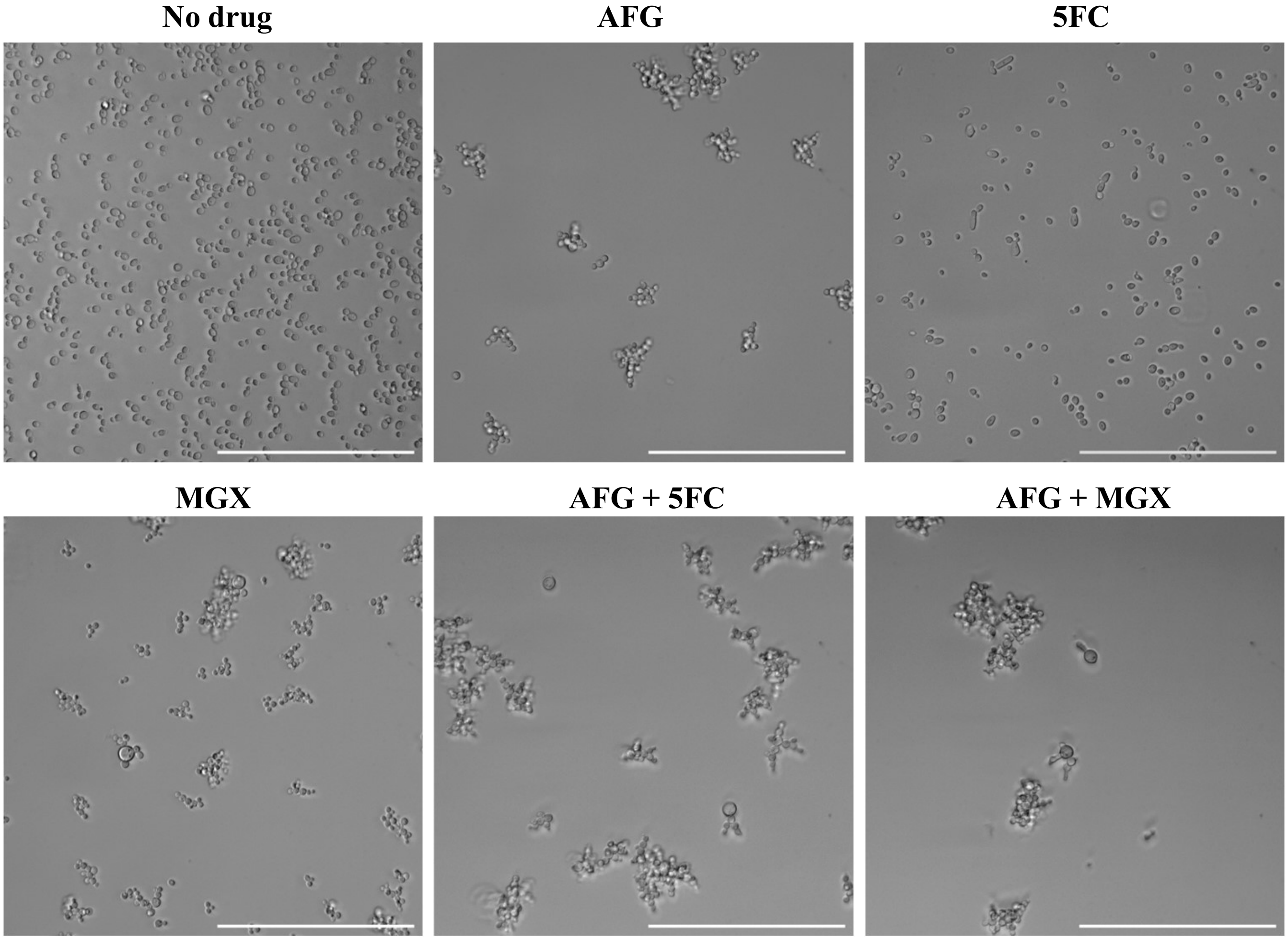

Figure S8. *C. auris* cell morphology after 24 h exposure to anidulafungin in combination with manogepix or 5-flucytosine.

Transmitted light images of *C. auris* B12663 cells grown in RPMI 2%G-MOPS with and without anidulafungin, manogepix or 5-flucytosine at 1x MIC_{50} in monotherapy and in combination for 24 h at 37 °C. Under exposure to anidulafungin, manogepix and both combinations the cells displayed a rounder morphology with the formation of aggregates, while 5-flucytosine treatment resulted in a more elongated phenotype. Additionally, enlarged, round cells were observed in the presence of manogepix and both combinations. Scale bar: 100 µm. 5FC, 5-flucytosine; AFG, anidulafungin; MGX, manogepix.

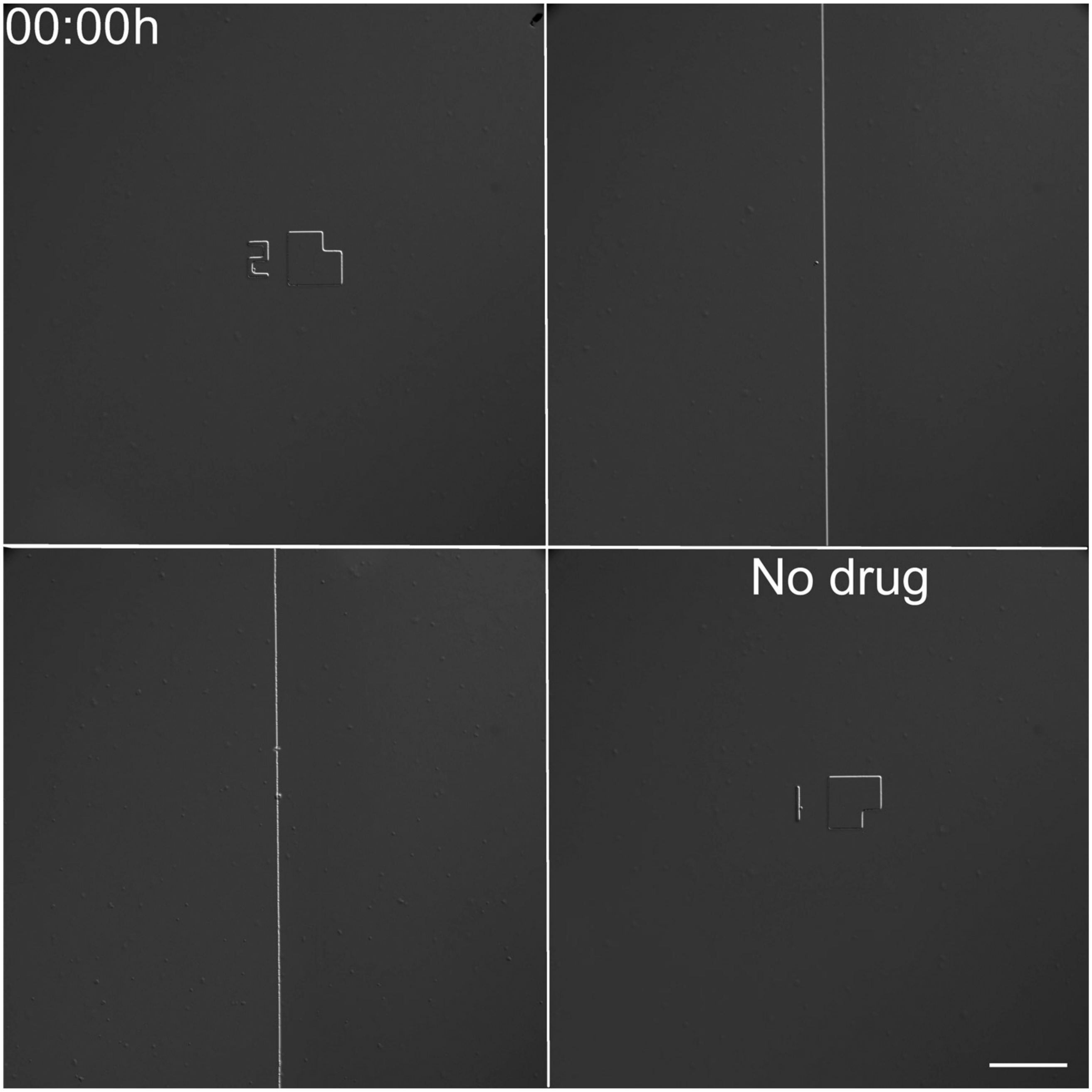

Movie S1. Exposure of *C. auris* to anidulafungin and 5-flucytosine for 24 h.

Microfluidics DIC imaging of *C. auris* B12663 cells grown in the presence of RPMI 2%G-MOPS for 4 h, followed by further RPMI 2%G-MOPS or treatment with anidulafungin and 5-flucytosine alone or in combination at their MICs for 16 h. Scale bar: 100 µm. 5FC, 5-flucytosine; AFG, anidulafungin.

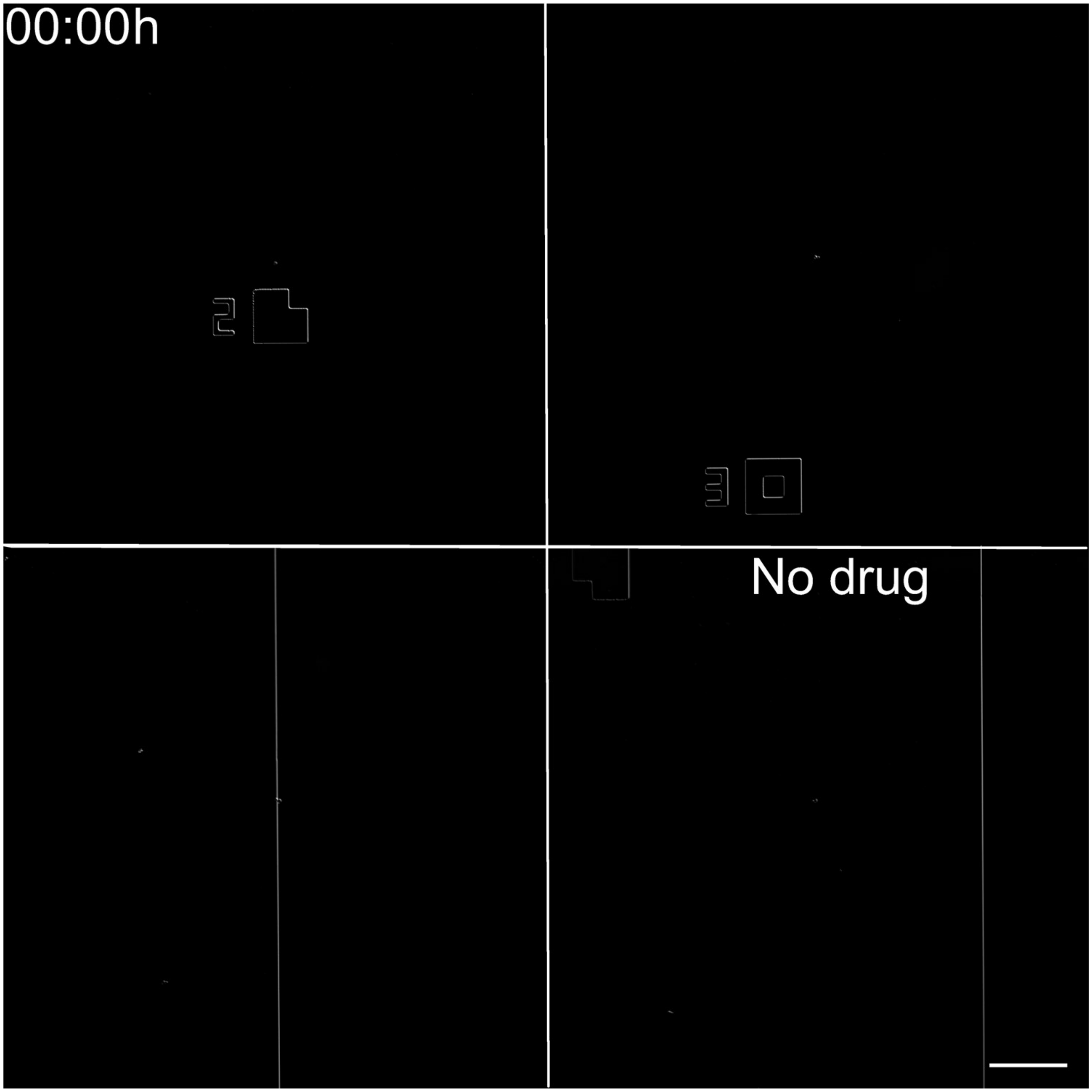

Movie S2. Exposure of *C. auris* to anidulafungin and manogepix for 24 h.

Microfluidics DIC imaging of *C. auris* B12663 cells grown in the presence of RPMI 2%G-MOPS for 4 h, followed by further RPMI 2%G-MOPS or treatment with anidulafungin and manogepix alone or in combination at their MICs for 16 h. Scale bar: 100 µm. AFG, anidulafungin; MGX, manogepix.
